# Effect of the Total Saponins of *Bupleurum chinense* DC. Water Extracts Following Ultrafiltration Pretreatment on Macroporous Resin Adsorption

**DOI:** 10.3390/molecules29215153

**Published:** 2024-10-31

**Authors:** Ruihong Wang, Hongbo Liu, Zhishu Tang, Huaxu Zhu, Huan Liu, Ran Guo, Zhongxing Song, Hongbo Xu, Bo Li, Guolong Li, Yue Zhang

**Affiliations:** 1 State Key Laboratory of Research and Development for Characteristic Resources of Qin Medicine (Cultivation), Co-Construction Collaborative Innovation Center for Chinese Medicine Resources Industrialization by Shaanxi & Education Ministry, Shaanxi University of Chinese Medicine, Xianyang 712083, China; ruihonwang@126.com (R.W.); 17691363394@163.com (H.L.); gr_0228@163.com (R.G.); szx74816@sina.com (Z.S.); 501018@sntcm.edu.cn (H.X.); 1501033@sntcm.edu.cn (G.L.); 2 Shaanxi University Engineering Research Center for the Research & Application of Membrane Technology for the Green Manufacturing of Traditional Chinese Medicine, Shaanxi University of Chinese Medicine, Xianyang 712083, China; 3 School of Chinese Materia Medica, Beijing University of Chinese Medicine, Beijing 102488, China; 4 Jiangsu Collaborative Innovation Center of Chinese Medicinal Resources Industrialization, Nanjing University of Chinese Medicine, Nanjing 210023, China; boli@njucm.edu.cn (B.L.); zhyue@njucm.edu.cn (Y.Z.); 5 Jiangsu Botanical Medicine Refinement Engineering Research Center, Nanjing University of Chinese Medicine, Nanjing 210023, China

**Keywords:** traditional Chinese medicine, ultrafiltration, adsorption, macroporous resin

## Abstract

Macroporous resin is an efficient separation technology that plays a crucial role in the separation and purification of traditional Chinese medicine (TCM). However, the application of macroporous resins in TCM pharmaceuticals is hindered by serious fouling caused by the complex materials used in TCM. This study examines the impact of ultrafiltration (UF) membrane technology on the macroporous resin adsorption behavior of TCM extracts. In this paper, *Bupleurum chinense* DC. (*B. chinense*) water extracts were included as an example to study the effect of UF pretreatment on the macroporous resin adsorption of total saponins. The study results indicated that the adsorption of total saponins constituents from the water extracts of *B. chinense* on the macroporous resin followed the pseudo-second-order kinetic model and the Langmuir model. The thermodynamic parameters of adsorption, including enthalpy changes and Gibbs free energies, were negative, while entropy changes were positive. These results demonstrated that the total saponin components form a monolayer adsorption layer by spontaneous thermal adsorption on the macroporous resin, and that the adsorption rate is not determined by the rate of intraparticle diffusion. Following treatment with a UF membrane with an average molecular weight cut-off of 50 kDa, the protein, starch, pectin, tannin, and other impurities in the water extracts of *B. chinense* were reduced, while the total saponin content was retained at 82.32%. The adsorption kinetic model of the saponin constituents on the macroporous resin remained unchanged and was consistent with both the second-order kinetic model and the Langmuir model; the adsorption rate of the second-order kinetic model increased by 1.3 times and in the Langmuir model at 25 °C, the adsorption performance improved by 1.16 times compared to the original extracts. This study revealed that UF technology as a pretreatment method can reduce the fouling of macroporous resin by TCM extracts and improve the adsorption performance of macroporous resin.

## 1. Introduction

Under the guidance of traditional Chinese medicine (TCM) theory, drugs used for disease prevention, diagnosis, treatment, and rehabilitation are referred to as TCM. Chinese medicines are natural products, including plant, animal, mineral, and some chemical and biological medicines. TCM is often plant-based, leading to the saying that ‘all medicines are herb-based’. TCMs contain active ingredients such as flavonoids, saponins, alkaloids and terpenoids, as well as other constituents from plant tissues such as proteins, starch, pectin, pigments and so on. These plant tissue components do not have specific biological activity and are often considered inactive ingredients or impurities. The presence of these components can increase the dosage and reduce the stability of TCM preparations; therefore, they need to be removed.

Macroporous resin is a significant type of polymer separation material. Macroporous adsorption resin is a type of polymer adsorption resin that does not contain exchange groups and has a macroporous structure. It has a good macroporous network structure and a large specific surface area and can selectively adsorb organic compounds in aqueous solutions through physical adsorption. It is commonly used for the separation and purification of target substances. As a hydrophilic polymer, it has high adsorption capacity for adsorbed molecules, relatively low cost, and easy regeneration. Its purification principle is based on differences in molecular weight, polarity, or shape between different molecules and solutions [1,2]. It has been widely applied in the fields of food [3,4,5], medicine [6,7,8], and the environment [9,10,11]. Currently, macroporous resin is being used to separate and purify components of TCM [12,13,14]. This is achieved by exploiting the difference in adsorption force between the resin and the active and impure components. However, the complexity of TCM extracts means that macroporous resin can adsorb a significant number of impurities alongside the active ingredients. This results in impurities occupying the adsorption sites of the resin, reducing the amount of active ingredients that can be adsorbed. Additionally, this exacerbates resin pollution, reduces its service life, and hinders its regeneration [15]. Therefore, during practical use, TCM extracts are often pretreated to remove impurities. This reduces fouling of the macroporous resin, enhances its adsorption performance, and increases its service life.

Membrane technology is an emerging separation technology, and compared to traditional methods, membrane technology offers advantages such as high efficiency, low energy consumption, and environmental safety. Membrane technology, including microfiltration (MF), ultrafiltration (UF), nanofiltration (NF), reverse osmosis (RO), and membrane distillation (MD) [16], has been utilized for the treatment of wastewater [17,18,19], water resource recycling [20], chemical separation [21], biopharmaceuticals [22,23,24], and TCM pharmaceuticals [25,26,27]. When dealing with complex materials, macroporous resin technology and membrane technology can be applied jointly. The materials are pretreated with membrane technology first, and then the macroporous resin technology is applied to reduce fouling and improve the separation performance. Li [28] and colleagues discovered that pretreating pharmaceutical solutions with microfiltration and UF can effectively reduce macroporous resin fouling, improve resin reusability, extend its service life, and reduce production costs. Zhang et al. [29] employed a purification process that combined macroporous resin with membrane technology to efficiently extract anthocyanins from wild blueberry fruits. This combined process resulted in an increase in the anthocyanin content of each batch of extract products (≥35%), which exhibited excellent activity and solubility. Zhou et al. [30] employed membrane–macroporous resin coupling technology to obtain tea saponins that were not only pure and light in color but also cost-effective and environmentally friendly. This technology has the potential to become a new standard for industrial production of tea saponin products. Authors such as Conidi [31] pretreated artichoke wastewater for decontamination using UF and NF processes and tested three different macroporous resins by adsorption/desorption to prepare purified phenolic fractions with high antioxidant activity. Chen et al. [32] also coupled macroporous resin with membrane technology using a bioreactor hybrid system for municipal wastewater treatment to mitigate pollution.

Chaihu, prepared from the dried roots of *Bupleurum chinense* DC. or *Bupleurum scorzoneraefolium* WILLD., was first recorded in the “Shennong Bencao Jing”. The genus *Bupleurum* includes approximately 200 species, many of which have been pharmaceutically used for thousands of years, mainly in Asia and Europe [33,34]. It is a perennial herbaceous plant, rarely an annual herbaceous plant [35], usually harvested from September to October. The main components of *B. chinense* include saponins, sterols, volatile oils, fatty acids, polysaccharides, alkynes, fatty oils, and other components such as flavonoids, lignans, and coumarins as well as numerous trace elements [36].

The adsorption behavior of macroporous resin is crucial for designing its structure as well as predicting and optimizing the adsorption process. However, there have been few studies on the changes in the adsorption behavior of macroporous resin after the feed solution undergoes pretreatment by UF. Understanding this is important for selecting the pretreatment method of the feed solution and optimizing the adsorption process of the macroporous resin. This thesis investigates the changes in adsorption kinetics and thermodynamics of a macroporous resin using the water extracts of *B. chinense* after UF treatment. The goal of this study is to provide a reference for the high-efficiency application of the membrane–macroporous resin coupling technology in the field of TCM.

## 2. Results and Discussion

### 2.1. Selecting the Appropriate Pretreatment UF Membranes

**Figure 1:**
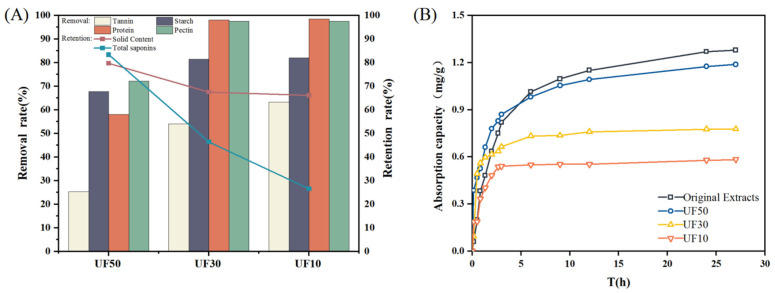
Retention of active ingredients and removal of impurities by different UF membranes (**A**); adsorption kinetic curves of total saponins in the original extracts, UF50, 30, and 10 (**B**).

UF is a low-pressure purification process that separates substances based on their membrane pore sizes and ionic charges. Figure 1A displays the retention of active ingredients and removal of impurities by different UF membranes, and Table 1 shows the properties of the water extracts of *B. chinense*. It can be seen that following pretreatment with different UF membranes, turbidity and viscosity are significantly reduced, while other numerical changes are not significant.

Further testing for total solid content, total saponin, and polymer impurities (protein, starch, pectin, and tannin) in each sample solution revealed a gradual decrease in the retention of each component with a reduction in the average molecular weight cut-off of the UF membrane. After pretreatment with UF50, total solid content and total saponins were retained up to 79.7% and 82.32%, respectively. However, only 67.53%, 66.13% and 46.46%, 26.57% were retained after passing through UF30 and UF10, respectively. Additionally, the macromolecular impurity components of protein, starch, pectin, and tannin were removed: The UF50 was able to remove 58.05% of the protein and 72% of the pectin. The removal rate of protein and pectin was very high at approximately 98% when using UF30. This may be due to the small pore size of the membrane. The protein and pectin macromolecules were hindered from passing through due to the membrane structure, resulting in their retention. Additionally, the surface of the membrane became covered, leading to clogging of the membrane pores and an increase in retention rate. The removal of tannin and starch improved as the membrane pore size decreased. The UF50 removed 25.29% of tannin and 67.73% of starch. In addition, *B. chinense*, as one of the most representative and important TMCs, has a very complex extract composition, including saponins, sterols, volatile oils, fatty acids, polysaccharides, coumarins, flavonoids, enzymes, fatty oils and other trace elements. The impurities also contain pigments, pectin, resin, starch, tannins, cellulose, proteins, and suspension of uncertain components. After UF, some impurities of large molecules were intercepted, but there were still many components that were not intercepted.

Figure 1B shows the curves of static adsorption capacity Q_t_ versus adsorption time of macroporous resin on the total saponins content of original extracts, UF50, 30, and 10. It can be seen that the static adsorption capacity of the original extracts was the highest, which was up to 1.27 mg/mL, and following pretreatment, the static adsorption capacity of the UF50 was up to 1.1884 mg/mL, and for the UF30 and UF10, the static adsorption capacity decreased further due to the decrease in total saponin, which was close to half of the original extracts. However, it can be seen from the curves that the static adsorption capacity growth rate was obviously much faster than that of the original extracts in the 4 h before adsorption with UF50 and UF30, indicating that pretreatment of the UF membrane can effectively reduce the impurities in the medicinal solution, which may be conducive to acceleration of the adsorption of the total saponins of *B. chinense* by the macroporous resin. In conclusion, UF50 pretreatment of the medicinal solution can effectively remove the impurity components while avoiding excessive loss of effective components, which was the optimal choice.

### 2.2. Adsorption Kinetics of Original Extracts and the UF50 on Macroporous Resin

**Figure 2:**
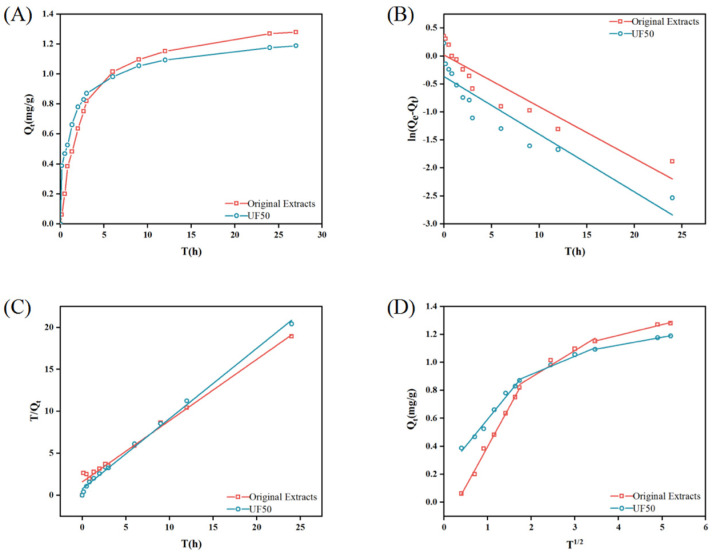
Adsorption time-course of total saponins on macroporous resin at 25 °C (**A**); linear correlations of the time-course data, based on the pseudo-first-order (**B**); pseudo-second-order (**C**) and intra-particle diffusion (**D**) models for total saponins on macroporous resin.

To evaluate the adsorption performance of macroporous resin for the adsorption of total saponins of *B. chinense* and the effect of UF membrane pretreatment on the adsorption behavior, comparative adsorption experiments were conducted at room temperature. As shown in Figure 2A, the adsorption capacity of the macroporous resin gradually increased over time, as shown by its adsorption kinetic curve. Within the first 4 h of adsorption, there were more available adsorption sites on the macroporous resin, resulting in a rapid increase in adsorption capacity. It was evident that the rate of total saponin adsorption by UF50 was significantly faster. This was because the pretreatment effectively removed impurities from the solution, reducing competition for total saponins in the macroporous resin adsorption process, which effectively increased the adsorption efficiency. The adsorption rate of both solutions between 4 and 12 h significantly decreased. This was due to the majority of adsorption sites on the resin being occupied. However, it should be noted that the UF50 reached adsorption equilibrium faster, which supports the previous viewpoint. After 12 h, the adsorbed amount of both solutions no longer changed and reached equilibrium. This was due to the lower initial saponin content in the UF50, resulting in a smaller static adsorption capacity at equilibrium.

To illustrate the adsorption mechanism, we used a pseudo-first-order model (Figure 2B), a pseudo-second-order model (Figure 2C), and an intraparticle diffusion model (Figure 2D). The correlation fitting parameters of the adsorption kinetics were determined (Table 2). Firstly, for the pseudo-first-order model, the correlation fitting parameters for the original extracts and the UF50 were 0.8694 and 0.8533, respectively, and the theoretical maximal adsorption Qe (1.0172 mg/mL, 0.6925 mg/mL) and the measured equilibrium static adsorption capacities (1.2789 mg/mL, 1.1884 mg/mL) showed a gap, which indicated that the adsorption of total saponins on macroporous resin was not a first-order reaction. The determination coefficients (R^2^) of the adsorption kinetic models for both sample solutions fitted the pseudo-second-order model (R^2^ = 0.9858, R^2^ = 0.9960, respectively) better, and Qe (1.3708 mg/mL, 1.1935 mg/mL) was closer to the measured equilibrium static adsorption capacity than the first-order model, which suggests that the pseudo-second-order model was able to better describe this adsorption process, and that adsorption processes are more than one factor (limited by factors such as adsorption site limitation, diffusion limitation and chemical reaction) and can be limited by two or more rates. On the other hand, the pseudo-second-order model of the UF50 had a greater rate constant K_2_ (0.4353) than the original extracts (0.3332), reaching 1.3 times that of the original extract, suggesting that UF promoted the adsorption of total saponins on the macroporous resin and reduced the interference of impurities.

In general, the adsorption process on porous adsorbents can be divided into four parts: transport of the adsorbed substance in solution; transport of the adsorbed substance on the porous adsorbent around the liquid; diffusion of adsorbed particles through the pores and channels of the adsorbent; and interactions between the active sites inside the adsorbent and the adsorbed substance [37]. As shown in Figure 2D, the adsorption of total saponins on macroporous resin (q_t_ versus $t^{\frac{1}{2}}$) was not a single linear, and the whole adsorption process could be divided into three stages: the first stage (0–3 h) was controlled by boundary diffusion (k_i_ 0.5698, 0.3821, respectively), with the highest adsorption rate. The second (3–12 h) belonged to the gradual adsorption process, in which the rate of intra-particle diffusion was limited (k_ii_ 0.1912, 0.1301, respectively). The third stage (12–24 h) belonged to the final gradual equilibrium stage (k_iii_ is 0.0766, 0.0561, respectively), and the rate of diffusion was the smallest [4,38]. Also, in the kinetic modeling parameter of intra-particle diffusion, the positive values of C, which can represent the thickness of the boundary layer, indicate that the straight line could not go through the origin [38]. This further suggested that the main rate limiting factor for the adsorption of total saponins was not just intra-particle diffusion. Wu et al. [39] obtained comparable results. As for the adsorption comparison between the two solutions, we found that the adsorption rate of the original extracts was greater than UF50 at all stages, and we speculate that this might be due to the UF process retaining some of the large particles, which reduced the diffusion of the particles in the adsorption process, and thus showed a smaller adsorption rate.

### 2.3. Adsorption Isotherms and Adsorption Thermodynamics

**Figure 3:**
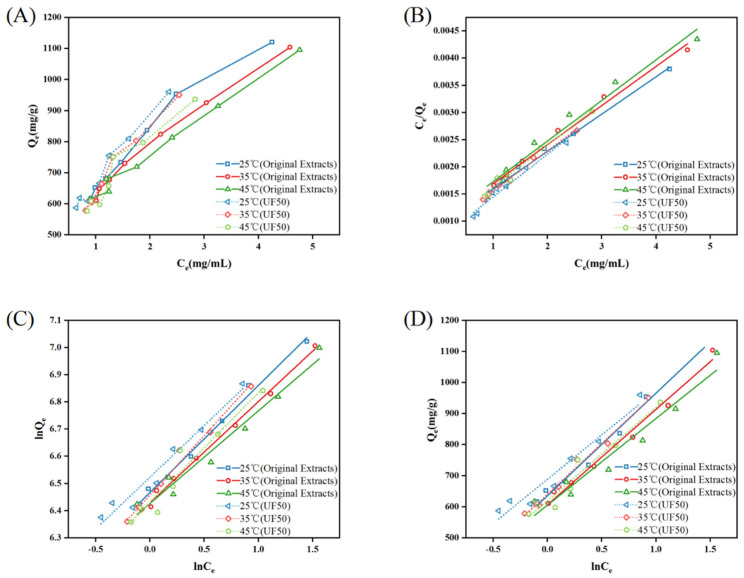
Adsorption isotherms for total saponins of the original extracts and “UF50” at 25, 35 and 45 °C. Plot of Q_e_ (equilibrium adsorption capacity of total saponins, in mg/g) against C_e_ (equilibrium of total saponin concentration in solution, in mg/mL) (**A**); linear correlations of the adsorption isotherm data based on the Langmuir (**B**), Freundlich (**C**) and Temkin (**D**) adsorption models.

The adsorption isotherms describe the relationship between the equilibrium concentration of an adsorbate in solution and the amount of solid adsorbent at a given temperature under adsorption equilibrium conditions [40]. As shown in Figure 3A, the adsorption capacity of total saponins on macroporous resin increased with an increase in the initial concentration, but this increasing trend gradually slowed down when the initial concentration increased. This may be because when the concentration of total saponins was low, there were a large number of adsorption sites on the macroporous resin, with less competition for adsorption and more complete adsorption. As the concentration increased, the competition for adsorption sites increased, the number of sites gradually decreased, and the increase in adsorption capacity slowed down. At the same concentration, as the temperature increased, the adsorption capacity of the resin for total saponins gradually decreased, indicating that high temperature is not conducive to the adsorption process. Zhou et al. [41] obtained similar results in their study. It was also observed that the adsorption trend of the UF50 at different temperatures was the same as that of the original extracts, indicating that the UF pretreatment did not change the adsorption mechanism of the resin for total saponins.

In order to more accurately describe the behavior of the resin adsorption process, linear fitting was performed on the isothermal adsorption process using the Langmuir model, Freundlich model, and Temkin model, as shown in Figure 3B–D. Table 3 shows the Langmuir model, which describes an adsorbent with a surface containing uniform adsorption sites, and neighboring molecules that do not interact [38]. The Langmuir model’s R_L_ (equilibrium constant) represents the practicality of the adsorption process of total saponins by macroporous resin. In this experiment, the R_L_ values were 0.996 and 0.997, (0 < R_L_ < 1) favorable, respectively, indicating that the total saponins are easily adsorbed by the macroporous resin. The Freundlich model implies that the adsorbent surface is uneven and molecules may interfere with each other. The total saponins also exhibited good accordance with the adsorption isotherms according to the values of the linear regression determination coefficients. The KF value (Freundlich constant) decreased with increasing temperature, indicating that low temperature is beneficial for the adsorption of resin [39]; 0 $<\frac{1}{n}<$ 1 also indicates that the total saponins are easily adsorbed by the macroporous resin [38]. The Temkin model is a mathematical model commonly used to describe the diffusion of substances within particles during adsorption processes. Temkin proposed a porous medium model in 1962 to describe the mass transfer process within solid particles, where K_T_ is an equilibrium constant that reflects the binding energy. In this experiment, the K_T_ value gradually decreased in the temperature range of 25 °C to 45 °C, indicating that the binding energy is weak at higher temperatures [38]. By comparing the three models, it can be seen that the Langmuir model had the highest R^2^ value compared to the Freundlich model and Temkin model, and best illustrated the adsorption performance of macroporous resin for total saponins.

Adsorption thermodynamics can elucidate the changes in energy in the system before and after the adsorption of saponin by the macroporous resin and determine whether the adsorption process is spontaneous. The thermodynamic parameters of the adsorption isotherm are shown in Table 4. The absolute value of ∆G was lowest at 25 °C, indicating that the spontaneity of the reaction was highest at low temperatures. ∆H<0 revealed that the reaction was an exothermic reaction, consistent with the adsorption isotherm results, and ∆H<40 kJ/mol indicated that the adsorption process was mainly physical adsorption. The macroporous resin mainly adsorbed total saponins through van der Waals forces (∆G<0, ∆H<0,∆S>0), indicating that regardless of temperature changes, the adsorption of total saponins on the resin can occur spontaneously [42]. Before and after adsorption, the degree of confusion in the system increased, indicating an increase in exothermic entropy.

## 3. Materials and Methods

### 3.1. Materials

The macroporous resin (D-101) was purchased from Xi’an Lanshen Special Resin Co., Ltd., (Xi’an, China). The roots of *Bupleurum chinense* DC. were acquired in October 2022 from Shaanxi Xingshengde Pharmaceutical Co., Ltd., (Xi’an, China). Polyethersulfone UF membranes (with the molecular weight cutoff of 50, 30, and 10 kDa) were purchased from Risingsun Membrane Technology (Beijing) Co., Ltd., (Beijing, China). An electric heating jacket (MH-3000) was purchased from Beijing Kewei Yongxing Instrument Company (Beijing, China). The HZQ-300AC temperature shaker was purchased from Shanghai Yiheng Scientific Instrument Co., Ltd., (Shanghai, China). The 8400 UF cup (XFUF0760) was purchased from Millipore Corporation (Billerica, MA, USA).

### 3.2. Preparation and Pretreatment of the Water Extracts of B. chinense

#### 3.2.1. Preparation of the Water Extracts of *B. chinense*

An appropriate amount of *B. chinense* was weighed and added to a round-bottomed flask; then, a 10-fold amount of distilled water was added, heated, and underwent condensation reflux extraction twice, for 2 h each time. This was followed by hot filtration and combination with the liquid. These were the original extracts of *B. chinense*, termed the “original extracts”.

#### 3.2.2. UF Pretreatment of the Water Extracts of *B. chinense*

The original extracts of *B. chinense* were filtered through the UF membrane with a molecular weight cutoff of 50, 30, and 10 kDa at 200 kPa pressure. The UF permeates obtained by different UF membranes were termed “UF50”, “UF30”, and “UF10”, respectively.

#### 3.2.3. Properties of the Original Extracts and UF50

Viscosity was measured using a Brookfield Viscometer (LVDV-II, Brookfield Engineering Labs, Inc., Middleboro, MA, USA). pH and conductivity were measured using a conductivity meter (Seven Excellence S400-K, Mettler Toledo, Switzerland). Turbidity was determined with a scattered light turbidimeter (WZS-188, NESA Scientific Instrument Co., Ltd., Suzhou, China). The total solid content was determined by placing a known volume of the sample in an evaporating dish and completely drying the sample in an oven at 105 ± 2 °C.

Determination of the total saponin content in *B. chinense* was performed using UV spectrophotometry [43]. Accurately, 0.2 mL of the test solution and control solution were aspirated, 0.1 mL of 1% p-dimethylaminobenzaldehyde ethanol solution was added, and the solution was heated in a 70 °C water bath for 10 min, and then removed and cooled; 4.0mL of phosphoric acid was added, reacted at 70 °C for 30 min, and left to stand at room temperature. The absorbance of the test solution and control solution was measured at a wavelength of 545 nm, using methanol as the blank solution. A standard curve was drawn using saikosaponin A as the reference solution.

The protein, starch, pectin, and tannin contents were determined as described in our previous work [44]. Briefly, protein determination was carried out using the Bradford method. Starch determination was carried out using the enzymatic hydrolysis method. The weight method was used for pectin determination: pectin was saponified to produce calcium gluconate and then acidified with acetic acid to produce calcium gluconate; calcium salt was added to precipitate calcium gluconate, dried, and weighed. The quantification of tannins was carried out using the skin powder weight method.

All tests were performed at room temperature.

### 3.3. Static Adsorption

For the pretreatment of macroporous resin, the macroporous resin was soaked in 80% ethanol for 24 h. The resin was then washed with pure water until no alcohol remained, dried in an oven at 60 °C, and set aside [28].

The original extracts and UF permeates of *B. chinense* (200 mL) were each placed in 250 mL conical flasks. Then, 2 g of the macroporous resin (dry weight) was added to each flask. The flasks were stoppered and adsorption was conducted at 25 °C and 60 rpm on a constant-temperature shaking table for 24 h. During the adsorption process, the content of total saponins and proteins in the supernatants was measured at specific time intervals ranging from 10 min to 24 h. A small sample was aspirated and analyzed for total saponins, total solid, protein, starch, pectin, and tannin. The static adsorption capacity [45] was calculated using the following formula:

(1) $$Q_{e}=\frac{\left({C_{0}-C}_{e}\right)V_{i}}{W}$$

where Q_e_ (mg/g) is the adsorption capacity value at adsorption equilibrium; C_0_ (mg/mL) and C_e_ (mg/mL) are the initial equilibrium concentration and equilibrium concentration of total saponins of *B. chinense* in the sample solution, respectively; V_i_ (mL) is the volume of the initial sample solution; and W (g) is the dry weight of the macroporous resin.

In order to understand the mechanism involved in the adsorption process, the above operation was repeated by selecting suitable UF permeates and the original extracts, and fitting using an adsorption kinetic model [46,47,48].

Pseudo-first order kinetic model:

(2) $$\ln\left(Q_{e}-Q_{t}\right)=\ln Q_{e}-K_{1}t$$

Pseudo-second-order kinetic model:

(3) $$\frac{t}{Q_{t}}=\frac{1}{K_{2}Q_{e}^{2}}+\frac{t}{Q_{e}}$$

Intraparticle diffusion kinetic model:

(4) $$Q_{t}=K_{3}t^{0.5}+C$$

where K_1_, K_2_, and K_3_ are the rate constants of the pseudo-first-order model, pseudo-second-order model and intraparticle diffusion model for the adsorption process, respectively, and the constant C (mg/g) represents the boundary layer diffusion effect. Q_e_ (mg/g) and Q_t_ (mg/g) are the adsorption capacity values at equilibrium and at various time periods.

### 3.4. Adsorption Isotherms and Thermodynamics

The total saponins of the original extracts and UF permeates were diluted to a range of concentrations (between 16.66–3.22 mg/mL and 11.56–2.38 mg/mL). Subsequently, 200 mL of the liquids with varying initial concentrations was mixed with 2 g of the macroporous resin (dry weight) and subjected to constant temperature shaking at different temperatures (25, 35, and 45 °C) and 60 rpm. Adsorption was carried out for 24 h, and the adsorption capacity at equilibrium was determined. The isothermal adsorption behavior was analyzed by fitting the adsorption isotherms using the Langmuir, Freundlich, and Temkin adsorption models [42]. The following equations were used to describe the adsorption isotherms:

Langmuir:

(5) $$\frac{C_{e}}{Q_{e}}=\frac{1}{K_{L}Q_{m}}+\frac{C_{e}}{Q_{m}}$$

The equilibrium constant R_L_ is expressed as:

(6) $$R_{L}=\frac{1}{1+K_{L}C_{0}}$$

where Q_m_ (mg/g) is the maximum adsorption capacity of the macroporous resin for total saponins, C_0_ (mg/mL) is the highest initial concentration of total saponins. C_e_ (mg/mL) is the equilibrium concentration of total saponins, K_L_ (mg/mL) is the Langmuir constant, and R_L_ is a dimensionless constant. The value of R_L_ gives an indication of the isotherm shape, which is either unfavorable (R_L_ > 1), linear (R_L_ = 1), favorable (0 < R_L_ < 1), or irreversible (R_L_ = 0) [39].

Freundlich:

(7) $$\ln Q_{e}=\frac{1}{n}\ln C_{e}+\ln K_{F}$$

where n and K_F_ are Freundlich constants; and n is related to the adsorption driving force and the energy distribution of the adsorption sites, and is a measure of adsorption strength or surface heterogeneity [42].

Temkin:

(8) $$Q_{e}=B_{T}\ln K_{T}+B_{T}\ln C_{e}$$

K_T_ is Temkin’s constant and B_T_ is a constant related to the heat of adsorption [38].

In addition, the study of adsorption thermodynamics helps to further analyze the adsorption mechanism of macroporous resin on total saponins of *B. chinense*, and UF permeates were compared to further analyze the adsorption behavior. The enthalpy change and entropy change Gibbs free energy change equations [38,49] are as follows:

(9) $$\ln K=-\frac{\Delta H}{RT}+\frac{∆S}{R}$$

(10) ∆G=−RTln⁡K

where Kc is the equilibrium partition coefficient, T is the absolute temperature (K), R is the gas constant (8.314 J·mol^−1^·K^−1)^), K is the adsorption equilibrium constant K_L_ in the Langmuir model, ΔH (kJ·mol^−1^), ΔS (J·mol^−1^·K^−1^) and ΔG (kJ·mol^−1^) are thermodynamic parameters.

## 4. Conclusions

In this study, taking *B. chinense* as an example, a UF membrane was used in the pretreatment of *B. chinense* extracts. The retention effects of different UF membrane pretreatments on *B. chinense* components were compared and analyzed, and UF50 was selected for subsequent research. The static adsorption kinetics and isothermal adsorption modeling of total saponins in *B. chinense* on macroporous resin before and after UF pretreatment were studied, as well as the effect of pretreatment on the adsorption mechanism. The adsorption of total saponins by macroporous resin conforms to the pseudo second-order model and Langmuir model and is not solely controlled by intraparticle diffusion. It is a spontaneous exothermic entropy increase process. The modeling results of UF pretreatment are consistent with the untreated results, indicating that UF pretreatment does not change the adsorption behavior of macroporous resin on total saponins. However, the improvement in its adsorption rate and the comparison of physicochemical parameters indicate that UF pretreatment can effectively reduce impurities in the drug solution, promote the adsorption of resin in the drug solution, and reduce pollution due to impurities on the resin in the drug solution. This provides a useful theoretical solution for combining UF pretreatment with resin adsorption in the future, to more efficiently separate effective ingredients of TCM and reduce resin pollution.

## Figures and Tables

**Table 1:** Properties of the water extracts of *B. chinense*.

Type	Viscosity (Pa.s)	Turbidity (NTU)	pH	Conductivity (μS/cm)	Total Solid (mg/mL)	Total Saponins (μg/mL)	Protein (μg/mL)	Tannin (μg/mL)	Starch (μg/mL)	Pectin (μg/mL)
original extracts	4.53 × 10^−3^	145.10	5.34	2932.86	207.60	25.00	404.00	1.51	43.20	724.00
UF50	1.46 × 10^−3^	4.91	4.97	2918.17	165.53	21.10	169.00	1.13	13.90	201.00
UF30	1.41 × 10^−3^	3.88	5.20	2780.23	140.19	11.70	7.92	6.93 × 10^−1^	8.01	18. 10
UF10	1.40 × 10^−3^	1.12	5.21	2679.48	137.29	6.70	6.48	5.53 × 10^−1^	7.74	18.0

**Table 2:** Adsorption kinetic equations and parameters of total saponin adsorption on macroporous resin.

Kinetics Model		Regression Equations	Parameters
Pseudo-first-order	original extracts	$\ln{(q}_{e}-q_{t})$ = −0.09230 t + 0.0171	k_1_ = 0.0923 qe = 1.017267 r^2^ = 0.8694
	UF50	$\ln{(q}_{e}-q_{t})$ = −0.1030 t − 0.3674	k_1_ = 0.103 qe = 0.692533 r^2^ = 0.8533
Pseudo-second-order	original extracts	$\frac{t}{q_{t}}$ = 0.7295 t + 1.597	k_2_ = 0.3332 q_e_ = 1.3708 r^2^ = 0.9858
	UF50	$\frac{t}{q_{t}}$ = 0.8379 t + 0.7486	k_2_ = 0.4353 q_e_ = 1.1935 r^2^ = 0.9960
Intra-particle diffusion	original extracts	$q_{t}$ = 0.56981 $t^{\frac{1}{2}}-$ 0.17159	K_1_ = 0.5698 C = −0.17159 r^2^ = 0.9939
		$q_{t}$ = 0.19121$t^{\frac{1}{2}}$ + 0.51116	K_1_ = 0.19121 C = 0.51116 r^2^ = 0.9440
		$q_{t}$ = 0.07655$t^{\frac{1}{2}}$ + 0.88705	K_1_ = 0.07655 C = 0.88705 r^2^ = 0.9828
	UF50	$q_{t}$ = 0.38209$t^{\frac{1}{2}}+$ 0.21184	K_1_ = 0.38209 C = 0.21184 r^2^ = 0.9845
		$q_{t}$ = 0.13006$t^{\frac{1}{2}}+$ 0.65378	K_1_ = 0.13006 C = 0.65378 r^2^ = 0.9788
		$q_{t}$ = 0.05612$t^{\frac{1}{2}}+$ 0.89869	K_1_ = 0.05612 C = 0.89869 r^2^ = 0.9967

**Table 3:** Equations and parameters of adsorption isotherms of total saponins.

Model		T (°C)	Equations	Parameters		
				K_L_	q_m_	R^2^
Langmuir	original extracts	25	$C_{e}/Q_{e}$ = 6.86$C_{e}$ + 9.21	0.7443	0.1457	0.9930
		35	$C_{e}/Q_{e}$ = 7.18$C_{e}$ + 9.17	0.7393	0.1392	0.9877
		45	$C_{e}/Q_{e}$ = 7.36$C_{e}$ + 9.99	0.7370	0.1357	0.9693
	UF50	25	$C_{e}/Q_{e}$ = 7.13$C_{e}$ + 8.26	0.8636	0.1400	0.9710
		35	$C_{e}/Q_{e}$ = 7.35$C_{e}$ + 8.67	0.8478	0.1360	0.9921
		45	$C_{e}/Q_{e}$ = 7.47$C_{e}$ + 8.94	0.8352	0.1337	0.9740
				K_F_	1/n	R^2^
Freundlich	original extracts	25	$\ln Q_{e}=$ 0.39$\ln C_{e}$ + 6.46	642.90	0.3941	0.9906
		35	$\ln Q_{e}$ = 0.37$\ln C_{e}$ + 6.42	619.55	0.3715	0.9945
		45	$\ln Q_{e}$ = 0.34$\ln C_{e}$ + 6.43	618.06	0.3410	0.9618
	UF50	25	$\ln Q_{e}$ = 0.38$\ln C_{e}$ + 6.52	680.61	0.3807	0.9544
		35	$\ln Q_{e}$ = 0.43$\ln C_{e}$ + 6.45	637.52	0.4378	0.9836
		45	$\ln Q_{e}$ = 0.40$\ln C_{e}$ + 6.42	619.36	0.4058	0.9268
				K_T_	B_T_	R^2^
Temkin	original extracts	25	$Q_{e}$ = 330.16$\ln C_{e}$ + 635.3	6.8504	330.16	0.9874
		35	$Q_{e}$ = 306.86$\ln C_{e}$ + 606.5	7.2191	306.86	0.9834
		45	$Q_{e}$ = 278.78$\ln C_{e}$ + 605.2	8.7673	278.78	0.9333
	UF50	25	$Q_{e}$ = 284.71$\ln C_{e}$ + 688.9	11.242	284.71	0.9416
		35	$Q_{e}$ = 325.92$\ln C_{e}$ + 640.7	7.1414	325.92	0.9875
		45	$Q_{e}$ = 299.81$\ln C_{e}$ + 619.8	7.9041	299.81	0.9382

**Table 4:** Thermodynamic parameters of macroporous resin for adsorption of total saponins.

	∆G (kJ/mol)			∆H (kJ/mol)	∆S (kJ/mol)
	25 °C	35 °C	45 °C		
original extracts	−154.712	−215.22	−275.728	−3.4412	6.0508
UF50	−179.663	−246.3	−312.936	−13.0721	6.6636

## Data Availability

Data are contained within the article.
